# Picture quiz

**Published:** 2017-08-07

**Figure:**
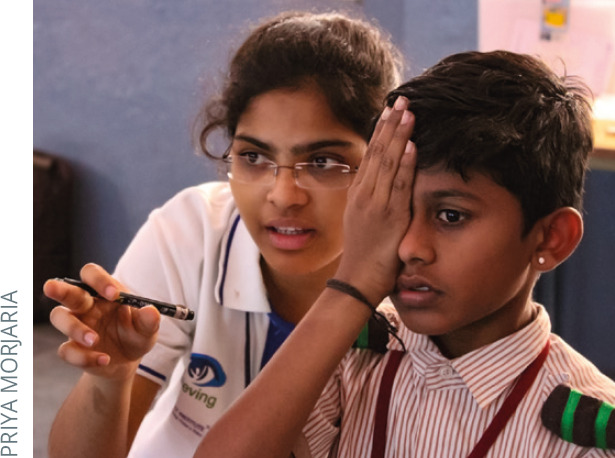


At school screening, an 8-year-old child is found to have presenting visual acuities of 6/6 in the right eye and 6/60 in the left.

**Tick ALL that are TRUE**

**Question 1 Which of the following conditions may be responsible?**
  □ **a**. Myopia
  □ **b**. Amblyopia
  □ **c**. Congenital cataract
  □ **d**. Toxoplasmosis
  □ **e**. Optic atrophy
**Question 2 What further tests are appropriate in this case?**
  □ **a**. Refraction
  □ **b**. Dilated fundus examination
  □ **c**. Corneal topography
  □ **d**. Cover test
  □ **e**. Ishihara test for colour blindness
**Question 3 Which of the following can be associated with visual impairment in a child?**
  □ **a**. Prematurity
  □ **b**. Family history of squint
  □ **c**. Maternal history of rubella infection
  □ **d**. Prolonged close work from an early age
  □ **e**. Photophobia
**Question 4 Amblyopia. Which statements are true?**
  □ **a**. Amblyopia may occur in a child with straight eyes
  □ **b**. Amblyopia is more commonly associated with short sight than long sight
  □ **c**. Unilateral cataract may cause amblyopia
  □ **d**. Severe astigmatism can cause bilateral amblyopia
  □ **e**. Unilateral congenital ptosis will not cause amblyopia

## ANSWERS

All may be responsible, except for optic atrophy, which would be more likely to affect both eyes. Myopia may be unilateral. Amblyopia may be due to unilateral hypermetropia or anisometropia. Congenital cataract in the left eye may have gone unnoticed until screening took place. A retinal lesion such as toxoplasmosis could also be responsible.
(a), (b) and (d). Refraction and a dilated fundus examination are essential to pick up refractive error or a retinal lesion. A cover test would confirm straight eyes or pick up a small squint that could be associated with amblyopia.
All may be associated with visual impairment in a child. Prematurity is a risk factor for ROP and myopia; squint and associated amblyopia are commonly inherited; maternal rubella is a risk factor for cataract; prolonged close work can induce myopia. Photophobia may be a sign of a condition leading to visual loss such as glaucoma or retinal dystrophy.
(a), (c) and (d) are true. Amblyopia is more commonly associated with long sight. Unilateral ptosis will cause amblyopia if the pupil is covered.

